# Obstetrician’s risk perception on the prescription of magnesium sulfate in severe preeclampsia and eclampsia: A qualitative study in Brazil

**DOI:** 10.1371/journal.pone.0172602

**Published:** 2017-03-16

**Authors:** Fátima Aparecida Lotufo, Mary Angela Parpinelli, Maria José Osis, Fernanda Garanhani Surita, Maria Laura Costa, José Guilherme Cecatti

**Affiliations:** 1 Department of Obstetrics and Gynecology, School of Medical Sciences of the University of Campinas, Campinas, State of São Paulo, Brazil; 2 Sociologist and Full Professor of the Postgraduate Program on Obstetrics and Gynecology–School of Medical Sciences of the University of Campinas, Campinas, State of São Paulo, Brazil; Johns Hopkins University, UNITED STATES

## Abstract

**Introduction:**

Magnesium sulfate (MgSO_4_) is the drug of choice for the prevention and control of seizures in the management of severe preeclampsia/eclampsia. Several barriers have been identified in the use of MgSO_4_, especially in low and middle-income settings.

**Objective:**

To describe the obstetrician’s perception on possible reasons for underutilizing magnesium sulfate to treat preeclampsia/eclampsia.

**Method:**

A qualitative clinical study, based on phenomenological reference by semi-structured interviews and open-ended discussions with obstetricians of the public healthcare system in primary care units (PCU) and referral maternity hospitals (RMH), in a southeastern Brazilian city.

**Results:**

Fear of drug toxicity was the major cause for not prescribing the medication in PCU. Fear was justified by insufficient technical, structural and organizational resources of healthcare facilities and by a shortage of physicians properly trained for adequate drug use.

**Conclusion:**

Fear of toxicity of magnesium sulfate was the main barrier towards timely and proper drug use. Periodic skill development and training of obstetricians, along with integration of the medical team in the work environment may contribute to decrease fear, ensuring safety of drug prescription and thus possibly reducing adverse outcomes related to PE.

## Introduction

Preeclampsia and eclampsia are among the three main causes of maternal death worldwide, affecting approximately 4.6% and 1.4% of all births, respectively [1,2]. Eclampsia is currently the main cause of maternal death in Brazil [3].

Magnesium sulfate (MgSO_4_) is one of the main interventions for the control and prevention of eclamptic seizures, with a good patient safety profile [4, 5]. The World Health Organization (WHO) has already acknowledged and recommended the use of magnesium sulfate as a strategy to reduce maternal morbidity and mortality [6]. There are two distinct drug regimens for the effective and safe use of magnesium sulfate, recommended by WHO and other international organizations. The first is given exclusively by the intravenous route (IV) (Zuspan, 1966) [7] and the second is a mixture of intravenous (IV) and intramuscular (IM) (Pritchard, 1955) [8] administration. The use of magnesium sulfate is allowed in a non-hospital setting, such as primary care facilities, especially considering the loading dose [9–11].

In Brazil, studies have reported high coverage of MgSO_4_, including 68% among women with severe maternal morbidity due to hypertensive causes [12], and 94% to 100% of women with eclampsia [13,14]. Although the use of the medication is extensive, maternal mortality from this cause still remains elevated in the country. The same has been shown in other studies worldwide, especially in low and middle income settings [14], suggesting that other barriers might be playing a role, such as inadequate time of drug prescription, lack of training, no evidence-based clinical protocols, and fear of toxicity [15–18].

Our group has previously conducted a general situational analysis on availability and use of MgSO_4_ for severe preeclampsia and eclampsia in the public health system and demonstrated that lack of processes integrating urgency/emergency care and specialized obstetric care possibly favors the inadequate use of MgSO_4_ [19]

The aim of this study was to explore the obstetrician’s perceived justification for underutilizing MgSO_4_ in severe preeclampsia/eclampsia.

## Materials and methods

### Study design

This was a qualitative clinical study using phenomenological methodology [20]. Experience was studied from a subject’s and subjective perspective [21–23], in order to reveal causal relationships between the phenomenon and the individual who gained the experience [24]. We respected the criteria consolidated in qualitative research (COREQ) [22].

Interviews were carried out until repetition of the phenomenon in statements also in maternity. Content saturation was achieved, based on frequency and intensity of certain aspects present during conversations, with the aims of the study in view. The research study took the experience presented and reproduced interviewee response. The context was considered, with rejection of analysis of right or wrong, faithful to segments of conversations, justifications, hypotheses, and feeling [25].

### Research setting and participants

For the current analysis, we considered the municipality of Campinas, in southeastern Brazil (city selected by convenience). Campinas has 1,080,034 inhabitants and 504,175 women of reproductive-age [26] with a 16,198 live born for the year 2015 according to the Municipal Department of Health [27]. The municipality also has good access to antenatal care (78.7% of pregnant women had seven prenatal visits or more in 2014) and childbirth care (99.6% of deliveries in the metropolitan region of Campinas occurred in hospitals [27]).

The healthcare system in Campinas provides coverage for an extended population of four and a half million inhabitants and is divided into five districts to facilitate planning and management (North, South, East, Southeast and Northeast), each with about 200,000 inhabitants. In each district, there is a planned surveillance in healthcare units and referral hospitals according to neighborhood and complexity. The city has 63 primary care units (PCU) and 3 referral maternity hospitals [28]. PCUs are responsible for primary healthcare with a well-defined territory and population [28]. Referral maternities are responsible for high-risk and emergency care during pregnancy, childbirth and postpartum period.

Both healthcare settings were chosen for the interviews: PCUs and referral maternity hospitals. Interviews were carried out by only one interviewer, trained in conducting a semi-structured interview with free flow of conversation.

Inclusion criteria for the study sample were obstetricians who worked in maternal care in the public healthcare system in the municipality of Campinas. A sample of obstetricians was taken from the PCU and maternity hospitals, who were willing to share their experiences, allowing a deep exploration of aspects relevant to the study. The 63 basic health units were randomized, in order to define a sequence of selected PCUs chosen for interviews with obstetricians, until saturation of content was achieved. A total of 14 PCU were visited, with one obstetrician interviewed per Unit; two referral Maternities were included, with a total of 16 interviews (10 from one maternity and 6 from the other).

### Data collection and analysis

Interviews were conducted during a 6-month period (July to December 2015). The researcher visited the selected PCU and referral maternity hospitals approaching only obstetricians in their working environment (there were no refusals to participation in any setting). For data collection, a semi-structured interview with open-ended questions and a triggering question was used. However, the interviewees were allowed to engage in free flow of conversation. Interviews were conducted in a private room at the workplace, lasting in average 20 minutes. Each obstetrician was asked to express his or her feelings about using MgSO_4_ in severe preeclampsia/eclampsia, thoughts on this experience and mechanisms to cope with these feelings.

Interviews were identified by letters and numbers indicating the location where they were held: primary care unit (PCU) and reference maternity hospital (RMH). The identity of the obstetricians and units visited remained confidential.

Interviews were recorded, guided by the triggering question, and transcribed by *Nvivo of QSR International*^®^ software. Transcriptions were assessed according to analytical technique of the Bardin content analyses. Initially, skimming and superficial reading of the conversations were performed. Ideas were identified for the response to research questions. The most commonly repeated contents or topics were selected for comparison and category construction [29, 30].

Data collected on sample characteristics were stored in *Excel*^*®*^ 2007 program. This database was analyzed for logical consistency and inconsistencies identified and further corrected. On descriptive analysis, the mean, median, standard deviation and minimum and maximum values were calculated for quantitative variables. Absolute (n) and relative (%) frequencies were also considered.

### Ethical approval

The study was conducted after approval of local IRB (State University of Campinas- CAISM) and the National Committee of Ethics in Research (Comissão de Ética em Pesquisa—CEP), under the letter of approval number 658.325. All principles regulating research on human beings according to the Brazilian Health Council (Resolution CNS 466/12) were respected, as well as the Declaration of Helsinki. A written informed consent was signed by all participants and their interviews were further recorded.

## Results

A total of 14 obstetricians from PCU and 16 from referral maternity hospitals were interviewed S1 Table. All had completed medical residency in Obstetrics and Gynecology with an average period of 18 to 19 years since graduation. The mean age of these obstetricians was 44 years Table 1.

**Table 1:** Characterization of obstetricians interviewed in PCU and maternity hospitals.

Characteristics of participants	PCU (n = 14)	MATERNITY HOSPITAL (n = 16)
Sex (n)		
Male	3	8
Female	11	8
Age in years ^a^	44.35 (31–60)	44,68 (32–62)
Residency in Obstetrics and Gynecology	14	16
Years since graduation ^a^	18.64 (7–35)	19.35 (7–30)

PCU: primary care unit.

^a^: mean (min–max).

**Fig 1:**
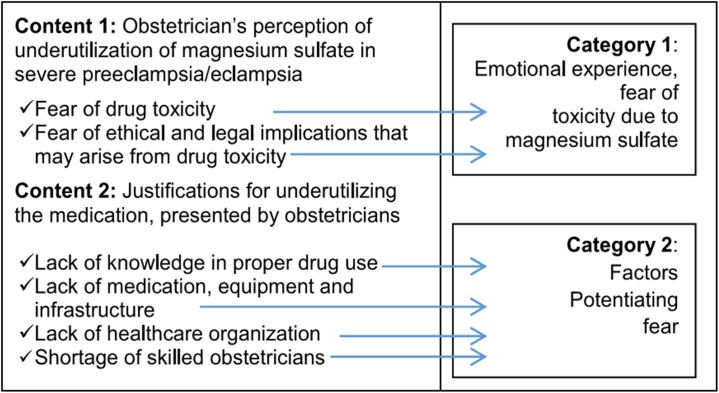
Process of category construction.

Two sensory categories were defined for analysis: emotional experience and potentiating factors. Emotional experience is a category that related to feelings of the obstetrician about the decision to prescribe magnesium sulfate in severe preeclampsia/eclampsia. In the category of potentiating factors, justifications for not prescribing the drug emerged. These factors were related to inadequate infra-structure, a shortage of material and equipment, difficulties in organizing healthcare and work processes in the PCU. Incorrect use of MgSO_4_ was also reported Fig 1.

The results are presented according to the constructed categories and workplace of study participants (PCU and referral maternity hospitals) at the time of the interviews.

### Emotional experience

Obstetricians interviewed in PCU believed that adverse events could occur during the use of MgSO_4_, and their greatest fear was cardiorespiratory arrest.

*“My greatest fear is* *respiratory arrest. I don’t know how to intubate”. **(PCU 5)***

*“We get really* *stressed out, afraid that something might happen, afraid of the adverse effects that may occur. Because here we have little resources, and this makes everything more difficult”. **(PCU 8)***

*“Because it causes toxicity,* *decreases respiratory rate and then we can lose her. If she dies, it will be our fault". **(PCU 11)***

Obstetricians felt unprepared to deal with complications that may arise from the use of magnesium sulfate that may trigger lawsuits.

*“I switched schedules* *so that I am not the only attending doctor in the unit, without an Intern Medicine clinician as support. It has been a long time since I did clinical practice. The Regional Medical Council makes demands on you… We are all alone”. **(PCU 7)***

*“You have to* *be very careful when filling out a medical chart, when it is time to take risks, because explanations will be required from you”. **(PCU 9)***

Concerning the experience of obstetricians working in a hospital, there seemed to be no fear in prescribing the drug, due to safety in a hospital environment perceived by these professionals. However, the interviewees in maternity hospitals recognized that prescription of magnesium sulfate generates a feeling of insecurity in the PCU.

*“No fear in using* *the medication here in the hospital. In the hospital the physician orders it, but the same professional would not administer the drug in a PCU due to lack of resources. **(M 10)***

*“Here we have* *no difficulty in dealing with this. Our team is highly trained.” **(M 11)***

*“I think it is the* *fear of managing cardiorespiratory arrest that is so frequently lectured in conferences: "Revert with gluconate in cardiorespiratory arrest". I think it is highlighted so often that it leads to fear”. **(M 8)***

*“Because although the* *medication is easily administered, there is a great myth about the issue of drug toxicity”. **(M 16)***

#### b. Potentiating factors

During interviews, potentiating factors for fear of prescribing magnesium sulfate were identified in obstetricians from the PCU and referral maternity hospitals. These factors could be associated with underutilization and/or incorrect use of the medication.

From an obstetricians’s perspective, limited knowledge and little experience in proper use of MgSO_4_ potentiated the fear of prescribing this drug.

*“The major* *difficulty is the lack of knowledge and fear of using the drug. Use it in the hospital with resources and equipment. In the PCU, its use is impossible”. **(PCU1)***

*“Since we do* *not use the medication very often, we even have to check the dose. We sort of know the amount, but need to review the exact dose” **(PCU 2).***

*“Fear of* *using medication due to complications, unfamiliarity with the sulfate”. **(PCU 7)***

*“The nursing* *staff receives intensive training and clinical update. This is practically nonexistent for the medical staff… You receive no clinical update”. **(PCU 9)***

Unsuitable material, equipment, and infrastructure were also cited by obstetricians from the PCU as justification for not using the medication in a proper and timely manner. However, in referral maternity hospitals resources seemed to be fully available.

*“Frequently* *there is no room available for the patient to rest. It is hard, because medication is lacking” **(PCU 1).***

*“I have nothing here. There* *is no backup, equipment. Nothing. No monitor, pulse oximeter, things that we need…There isn’t even oxygen here” **(PCU 3)**.*

*“Here in the* *Healthcare Center, if we need to prescribe magnesium sulfate, first thing needed would be the medication, which is missing here.” **(PCU 4)***

*“We don’t* *have the physical structure, we don’t even have epinephrine, we have nothing”. **(PCU 11)***

*“Here we have the* *resources, but not a lot is needed. The medication and a monitor are required.” **(M 13)***

*“There is no* *difficulty in dealing with this here, since we have the resources”. **(M 11)***

The organization of medical work in the PCU, specifically previous elective schedules and the limited working period of these units (from 8 to 14 hours), were potentiating factors for fear of using the medication:

*“I believe that* *I don’t need to prescribe sulfate here, because you need to monitor the patient frequently and here we cannot do this. I see other patients. I cannot stop caring for others to care for this patient. It is not possible…”. **(PCU 4)***

*“Here we have working hours,* *with a time to open and a time to close. I work shifts with a time to enter and a time to leave. When my shift is over, I want to get out. Once I waited until 8:30 pm for the emergency mobile unit to arrive” **(PCU 9).***

*“First, we would* *have to cancel patient schedule. Other patients would not understand. What I mean is that we need to keep a close look at the patient. There is no other way to do it”. **(PCU 13)***

## Discussion

Our results identified that fear was the main emotional experience described by obstetricians interviewed in PCUs. These healthcare workers withheld use of MgSO_4_ in severe preeclampsia/eclampsia motivated by the risks of adverse drug events in a severely ill patient. Along with the feeling of fear, other conditions revealed as concrete justifications for withholding drug prescription were considered potentiating factors, e.g. unfamiliarity with the drug and its correct use, shortage of material, equipment and infrastructure, and deficient healthcare organization in PCUs.

Despite evidence supporting that MgSO_4_ is the drug of choice for severe preeclampsia/eclampsia [9], and studies in low-income and middle-income countries, such as Brazil, where use of MgSO_4_ in eclampsia is highly prevalent, maternal mortality rates from these disorders remain elevated [13, 14]. The apparent paradox may derive from incorrect drug use as revealed by this study. It was observed that obstetricians working in PCU do not prescribe the medication, but transfer this responsibility to professionals in maternity hospitals. Drug prescription may be too late, worsening patient prognosis, along with none integrated healthcare between primary facilities and referral centers [19].

Severe adverse events due to the use of MgSO_4_ are rare. Respiratory arrest, which is the most feared event, occurred in 1% of all women with preeclampsia treated with MgSO_4_, establishing a high level of safety in clinical drug use [9]. Despite these rare events, some studies searched for the ideal minimum therapeutic dose. Assessment of serum magnesium concentrations by the Zuspan and Pritchard drug regimens, Salinger et al. found that minimum dose levels were much lower than those described as therapeutic [31]. Alternative low-dose regimens have been studied and may be as safe and efficient as the current standard regimen. These low doses of MgSO_4_ may contribute to a decreased perception of toxicity risk, increasing proper and timely drug use. However, until scientific evidence is established, recommendations propose maintenance of standard regimens [32].

Another concern about the safety of MgSO_4_ administration is vigilant monitoring during its use. The literature has sufficient evidence showing that clinical control is safe and the drug may be administered in primary care [5, 33]. Despite these recommendations, in our study we found that the lack of oximeter, multiparametric monitor, and other devices precluded MgSO_4_ prescription in the PCU. On the other hand, it was relevant that obstetricians also perceived a shortage of attending professionals. Patient monitoring, which was not delegated to other health professionals (nursing staff), became more difficult.

It is also necessary to recognize that lawsuits are among the aspects related to fear of drug prescription. Health professionals attempt to themselves by seeking more technological resources, referrals to other specialists and refusing to accept more critical cases. Those professionals avoid the responsibility for MgSO_4_ use, particularly in locations with low technological density [33].

In Brazil, the fear of lawsuits has increased among physicians and this defensive practice has become increasingly more common [34, 35]. Evidence-based medicine opposes defensive medicine, since it guides clinical practice in a rational and updated manner. When accessible to the physician, it becomes a powerful management tool to improve healthcare quality and thus reduce health impairment [36]. Access to evidence-based rational medicine requires continuous update and insertion of this knowledge into daily clinical practice. Fear revealed by the doctor and the supposed adoption of defensive practice were related to the lack of training for the management of obstetric emergencies. Training is currently not guaranteed by health administrations.

The development of professional skills, as well as improvement in physical structure, material/equipment and primary care organization, lead to qualified clinical care. Safety in the diagnosis and treatment of maternal emergencies is enhanced, including MgSO_4_ for management of severe preeclampsia/eclampsia. These components have also been identified by others in low-income and middle-income countries [37, 38] with similar results.

The work process of the obstetrician, his feelings, perceptions and experiences should be understood by investigating daily practice and dynamics when a diagnosis of severe preeclampsia/eclampsia is made. Failure to prescribe MgSO_4_ may lead to actions that alter behavior. Management of hypertensive emergencies could be improved, contributing to a reduction in maternal mortality.

Our study has limitations. It is not completely generalizable, since it only referred to the population studied in a specific workplace in the southeast of Brazil. On the other hand, the feelings identified in this study could possibly be experienced by other physicians in settings that are similar to ours, or even with more severe limitations.

## Conclusions

Obstetricians reported fear of prescribing MgSO_4_ in the PCU, based on the risks of severe adverse events. In general, justifications for having fear were related to unavailability of appropriate material, equipment/ infrastructure, and the way maternal healthcare is organized in the context of action. Skill development planning and periodic obstetrical training, especially considering safety of the drug, in addition to integration of the medical team in the work environment may contribute to reduce fear, broadening timely prescription and thus improving quality of maternal healthcare.

## Supporting information

S1 Table Complete data file on characterization of obstetricians interviewed in PCU and maternity hospitals. (XLSX)
